# Study of antimicrobial activity and mechanism of vapor-phase cinnamaldehyde for killing *Escherichia coli* based on fumigation method

**DOI:** 10.3389/fnut.2022.1040152

**Published:** 2022-10-31

**Authors:** Xuejuan Duan, Dongying Qin, Hongming Li, Tong Zhang, Yali Han, Yu qiang Huang, Dong He, Kegang Wu, Xianghua Chai, Chun Chen

**Affiliations:** ^1^School of Chemical Engineering and Light Industry, Guangdong University of Technology, Guangzhou, China; ^2^School of Biomedicine and Pharmaceutical Sciences, Guangdong University of Technology, Guangzhou, China; ^3^School of Food Science and Engineering, South China University of Technology, Guangzhou, China

**Keywords:** cinnamaldehyde, *Escherichia coli*, vapor-phase, antimicrobial activity, mechanism

## Abstract

The vapor-phase antibacterial activity of essential oils makes them suitable for applications in air disinfection and other fields. At present, vapor-phase antibacterial activity of plant-based essential oils has rarely been reported. Herein, we report a new approach to investigate the antimicrobial activity and mechanism of vapor-phase cinnamaldehyde using *Escherichia coli* (*E. coli*) and three other pathogenic bacteria (*Pseudomonas aeruginosa, Salmonella, Staphylococcus aureus*) as model bacteria. Plate fumigation and agar block transfer techniques were used to determine the antimicrobial activities of vapor-phase cinnamaldehyde fumigation on the four types of bacteria, and the mechanism of action was determined by electrical conductivity (EC), OD_260nm_ measurement, transmission electron microscopy (TEM), Fourier transform infrared spectroscopy (FTIR), and fluorescence spectroscopy. Cinnamaldehyde had good vapor-phase antibacterial activity against the four types of bacteria. The TEM, EC, and OD_260nm_ measurements showed that after fumigation with cinnamaldehyde, the ultrastructures of the cells were damaged, and plasmolysis, cell collapse, and leakage of intracellular substances were observed. The FTIR and fluorescence spectroscopy analyses showed that the secondary and tertiary structures of bacterial membrane proteins were altered. These findings indicate that the cell membrane is an important target for plant-based essential oils to exert their vapor-phase antimicrobial effects. The results showed that plant-based essential oils can be developed as volatile broad-spectrum disinfection products and vapor-phase antiseptics.

## Introduction

*Escherichia coli (E. coli)* is a common food contaminant that can causes food spoilage and poses severe threats to human health (1). In particular, the Shiga toxin-producing *E. coli* is a foodborne pathogen that can cause severe disease symptoms and death in humans (2). *E. coli* is also an airborne environmental pathogen in human habitats and animal breeding environments (3). Thus, it is important to eliminate *E. coli* and other similar pathogens in the air to prevent and control infectious diseases. At present, most environmental and food pathogens are eliminated using chemical disinfectants and antiseptics. However, these chemical substances may endanger human health and pollute the environment (4). Therefore, it is of great significance to identify new natural antibacterial agents with high efficiency and environmental protection for human health, dwelling environment, and livestock and poultry breeding.

The volatile antimicrobial activity of plant essential oils on the germs in food and air has garnered increasing attention. Cinnamon is abundant in China and has traditionally used as an antibacterial and antiseptic material, where its constituent cinnamon essential oil plays a major role. Cinnamaldehyde is the main component of cinnamon essential oil (5), and is a broad-spectrum antimicrobial agent that exerts potent inhibitory effects against *E. coli, Salmonella sp., Staphylococcus aureus*, and *Bacillus sp*. in liquid or solid phase (6–10). Cinnamaldehyde also has natural advantages in sterilizing and purifying pathogenic bacteria in the air owing to its own volatility. Inouye et al. (11) reported that vapors of aldehyde constituents like citral and cinnamaldehyde were most potent. Yun et al. (12) evaluated the antimicrobial activity of cinnamaldehyde as a gaseous treatment to reduce *Salmonella enterica serovar Typhimurium in vitro* and on tomatoes, the vitro tests showed that cinnamaldehyde had good inhibition of *Salmonella*. Houdkova et al. (13) reported that cinnamaldehyde possessed moderate antibacterial activity in vapor phase with MIC 256 μg/mL. Thus, cinnamaldehyde could be developed as a green environmental disinfectant and food vapor-phase antiseptics.

Several studies have investigated the antibacterial activity and mechanism of cinnamaldehyde (14–19), however, there are few studies on vapor-phase antibacterial mechanism. In order to verify the broad-spectrum antibacterial activity of cinnamaldehyde, *E. coli* and three other pathogenic bacteria (*Pseudomonas aeruginosa, Salmonella, Staphylococcus aureus*) was used as model bacteria to study the vapor-phase antibacterial activity of cinnamaldehyde by the plate fumigation and agar block transfer methods, and the vapor-phase antibacterial mechanism of cinnamaldehyde against *E. coli* was studied using transmission electron microscopy (TEM), infrared spectroscopy, Fourier transform infrared spectroscopy (FTIR), electrical conductivity (EC) and OD_260nm_ values. This research has very important theoretical guiding significance for the application of plant-based essential oils in various fields.

## Materials and methods

### Materials

*Escherichia Coli* (*ATCC8739*), *Pseudomonas aeruginosa* (ATCC9027), *Salmonella* (ATCC14028), *Staphylococcus aureus* (ATCC6538) was purchased from Guangdong Institute of Microbiology (Guangdong, China). Cinnamaldehyde (99%, Analytical purity) was supplied from Guangzhou xiangsixinqing technology Co., LTD, (Guangzhou, China). Nutrient agar was purchased from Guangdong Huankai Microbial Science and Technology Company Limited (Guangzhou, China). Copper grid (AGH100) was obtained from Head Biotechnology Company Limited (Beijing, China). Ethanol was obtained from Damao Chemical Reagent Factory (Tianjin, China). This bacterium was grown in nutrient agar (NA, Huankai, Guangzhou, China) for 24 h at 37°C. The PBS buffer is 0.05mol/L, pH 7.4. Other chemicals used were all of analytical grade.

The formula of phosphate buffer saline (PBS) (pH7.4, per 100 ml): NaCl 0.85 g, KCl 0.02 g, Na_2_HPO_4_**·**2H_2_O 0.113 g, NaH_2_PO_4_**·**12H_2_O 0.285 g, KH_2_PO_4_ 0.027 g.

### Strain activation and preparation of the bacterial suspensions

The bacterial strains were inoculated onto nutrient agar (NA). After 24 h of activation, the culture was delineated to separate the individual colonies on the NA. Then the single colonies were diluted with sterilized deionized water into 0.5 McFarland's standards for bacterial suspension.

### Bacterial activity of cinnamaldehyde vapor-phase fumigation

The experiment was performed using plate fumigation and agar block transfer (20). Bacterial suspension (40 μL) was added to the solidified NA medium and spread evenly with coating rods. The different concentration of cinnamaldehyde diluted with propanediol were added to the Petri dish cover (0.40 mL each) such that the space concentrations of the Petri dish were 1, 0.5, 0.25, 0.125, and 0.0625 μL/mL. The Petri dishes were sealed using sterile adhesive tape and then inverted and cultured at 37°C for 24 h. The lowest concentration of cinnamaldehyde in plates with no bacterial growth was considered as the MIC. Agar blocks (6 mm diameter) made by sterile hole punch from non-bacterial growth plates were transferred to the center of fresh NA plates (the bacterial side of the blocks were attached to the plates). The plates were then incubated at 37°C for 24 h to observe the growth of the bacterial plaque. The lowest concentration corresponding to the plate where the plaque did not grow was the MBC. Groups without cinnamaldehyde treatment were used as controls.

### Transmission electron microscopy

The effect of cinnamaldehyde on the bacterial cell wall integrity was determined by TEM following the method described by Duan et al. (20). Cinnamaldehyde (1 MIC, 2 MBC) was added to the Petri dish cover for fumigation (37°C for 24 h). Groups without cinnamaldehyde treatment were used as controls. The bacteria were collected in a 1.5 mL centrifuge tube and fixed in 200–400 μL of 2.5% glutaraldehyde at 4°C for 6 h. After fixation, the samples were washed thrice with PBS and centrifuged after each wash. After centrifugation (9,386×*g*, 3 min), the supernatant was finally discarded. Gradient dehydration was carried out for 10 min by serially adding 200 μL of 30, 50, 70, 95, and 100% ethanol to the sample. The centrifugation was repeated, and the supernatant was discarded. The bacteria were dispersed and resuspended in absolute ethanol. Thereafter, 1–2 drops of the bacterial suspension were added to the mesh copper grid and dried. Morphologies of the samples were observed by field emission transmission electron microscope (Talos F200S, Fei, Czech Republic).

### Electrical conductivity

Following Wu et al. (21), after incubation of *E. coli* at 37°C for 24 h, 0.4 mL of different concentrations of cinnamaldehyde were dispensed into the dish lid to achieve different concentrations (0.0625, 0.125, 0.25, 0.5, and 1 μL/mL, 24 h) in space, or the *E. coli* was fumigated at different times (0.25μL/mL). After fumigation, plate colonies were eluted with PBS. Each bacterial suspension was adjusted to OD_600nm_ 1.0 ± 0.01 (UV spectrophotometer TU-1950, Beijing Purkinje General Instrument Company), and the EC was measured using an EC meter (DDSJ-308A, Shanghai Yidian Scientific Instruments Co., Ltd.).

### The value of OD_**260 nm**_

Following Wu et al. (21), after incubation of *E. coli* at 37°C for 24 h, 0.4 mL of different concentrations of cinnamaldehyde were dispensed into the dish lid to achieve different concentrations (0.0625, 0.125, 0.25, 0.5, and 1 μL/mL, 24 h) in space, or *E. coli* was fumigated for different times (0.25μL/mL). After fumigation, the colonies were eluted with sterile distilled water to obtain bacterial suspensions. Each suspension was adjusted to OD_600nm_1.0 ± 0.01. After centrifugation (9,386×*g*, 3 min), 3 mL of the supernatant was retrieved and the absorbance at 260 nm was determined.

### Fourier transform infrared spectrometer

According to Duan et al. (20), bacteria colonies from plates treated with different concentrations of cinnamaldehyde were collected in 10 mL of PBS and centrifuged at 9,386×*g* for 10 min at 4°C. After centrifugation, the supernatant was discarded. The cells were washed twice with PBS and centrifuged at 9,386×*g* for 10 min at 4°C. After discarding the supernatant, the cells were vacuum freeze-dried (vacuum freeze-dryer, Scientz-18ND, Ningbo Xinzhi). A small amount of bacteria powder was combined with KBr pellets. Samples of the pellets were scanned using FTIR (Nicolet 6700, Thermo Fisher Scientific) in the spectral range of 400–4,000 cm^−1^. The FTIR spectra were obtained at a resolution of 2 cm^−1^.

### Fluorescence spectrophotometer

According to Wu et al. (22), after incubation of *E. coli* at 37°C for 24 h, 0.4 mL of cinnamaldehyde at different concentrations was dispensed into the dish lid to achieve concentrations of 0.0625, 0.125, 0.25, 0.5, and 1 μL/mL. A blank control plate was prepared simultaneously. The plate colonies were fumigated for 24 h, eluted with 10 mL of PBS, and centrifuged at 9,386×*g* for 10 min at 4°C. The supernatant was discarded and the bacteria were suspended in PBS. Each bacterial suspension was adjusted to an OD_600nm_ of 0.4 ± 0.02, and the fluorescence emitted by the amino acid residues in the bacterial suspensions was measured with a fluorescence spectrophotometer (FluoroMax-4, HORIBA, Jobin Yvon, Inc. USA). The emission spectra of the samples were scanned within 300–500 nm and the slit width for the excitation and emission spectra grating was 5 nm.

### Statistical analyses

The experiments were repeated thrice for each sample. The experimental data were analyzed and plotted using Origin 8.1, Omnic, and PeakFit. The results are expressed as the mean ± standard deviation. Infrared data were analyzed and processed according to Duan et al. (20).

## Results

### The antimicrobial activity of cinnamaldehyde in vapor-phase

The results of the MICs and MBCs of cinnamaldehyde against four types of bacteria are summarized in Table 1. The MICs of cinnamaldehyde vapor-phase fumigation of *E. Coli, Pseudomonas aeruginosa, and Staphylococcus aureus* were 0.25 μL/mL, while *Salmonella* was 0.125 μL/mL. The MBCs of cinnamaldehyde vapor-phase fumigation of *E. Coli, Salmonella and Staphylococcus aureus* were 0.5, 0.25 and 0.5 μL/mL respectively, which are higher than the MIC values. The MBC of cinnamaldehyde against *Pseudomonas aeruginosa* was >1 μL/mL. Thus, cinnamaldehyde had good antibacterial activity against four kinds of bacteria, and the MBC values of cinnamaldehyde against bacteria was higher than MIC values in the study.

**Table 1:** The antibacterial effect of cinnamaldehyde in vapor phase.

**Pathogenic bacterium**	**MIC (μL·mL^−1^)**	**MBC (μL·mL^−1^)**
*E. coli*	0.25	0.5
*Pseudomonas aeruginosa*	0.25	>1
*Salmonella*	0.125	0.25
*Staphylococcus aureus*	0.25	0.5

### Morphological analysis of cinnamaldehyde fumigation damage to *E. coli*

**Figure 1:**
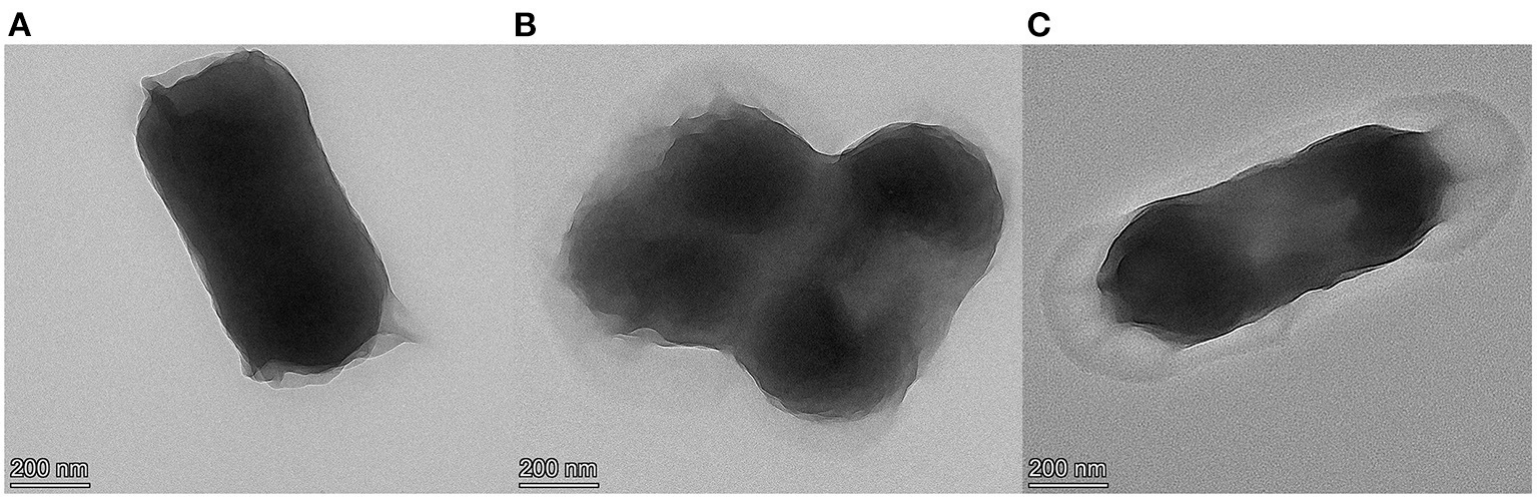
TEM micrographs of *E. coli*, untreated **(A)**, treated with cinnamaldehyde at MIC **(B)**, treated with cinnamaldehyde at 2MBC **(C)**.

**Figure 2:**
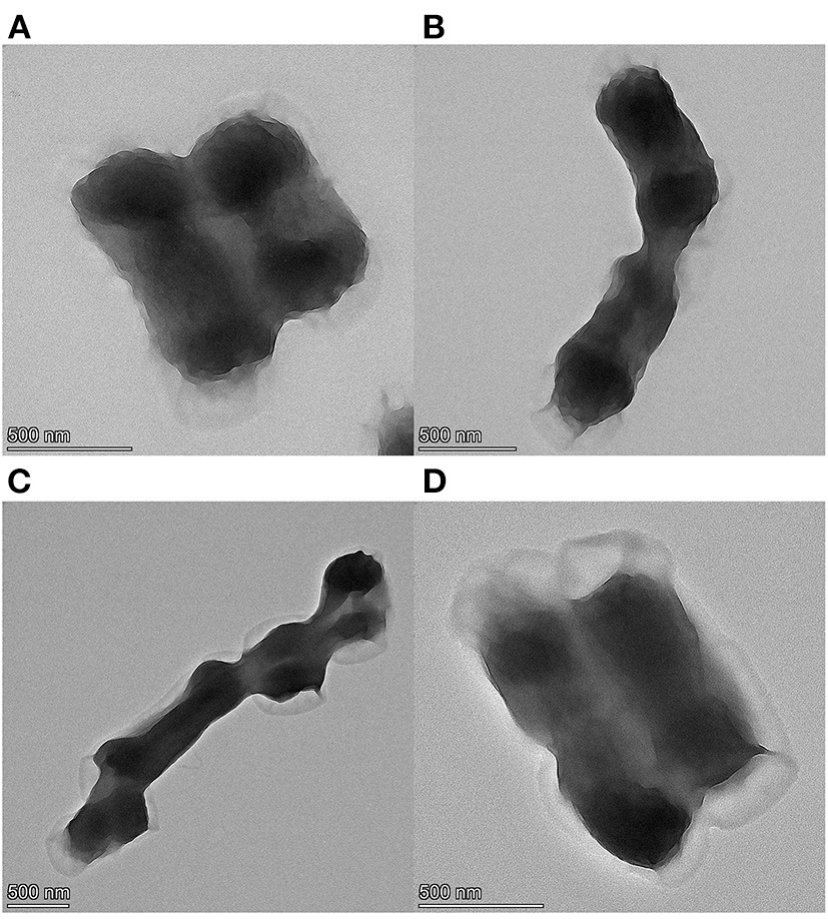
TEM micrographs of *E. coli*, **(A–D)** were treated with cinnamaldehyde at 2MBC for 3, 6, 9, 12 h, respectively.

The TEM results are shown in Figures 1, 2. Figure 1A shows the TEM image of untreated *E. coli*. The cell surface of *E. coli* without cinnamaldehyde treatment appeared smooth, the cells were plump, intact and without damage, and the contents were homogenous and dense. Figures 1B,C show the TEM results for *E. coli* treated with cinnamaldehyde fumigation at 1MIC and 2MBC, respectively. The surface of the *E. coli* appeared wrinkled and shrunken. Cellular dehydration, which leads to cytoplasmic aggregation, was also observed. Plasmolysis was evident and intracellular cavities were observed. Figure 1C shows the damage to the cell wall. Therefore, fumigation with cinnamaldehyde could work on the cell wall of *E. coli* to cause morphological changes in the cell surface, as well as damage the cell membrane of *E. coli* to induce intracellular contents. Generally, cinnamaldehyde causes cellular membrane damage, thereby affecting the normal physiological activity of the bacterium and ultimately leading to its death. Cinnamaldehyde is hydrophobic and can permeate the cell wall, thereby disrupting the cytoplasmic membrane structure (20). Based on the TEM results, cinnamaldehyde altered the cell surface morphology and disrupted the cell membrane, and studies by others (23) have shown that a slight change in the structure of the cell membrane could lead to changes in cellular physiological and metabolic activities, which in turn may inhibit cell growth or death. Figure 2 shows the TEM image for *E. coli* treated 3, 6, 9, and 12 h with cinnamaldehyde fumigation at 2MBC. The surface of the *E. coli* appeared wrinkled and shrunken. Plasmolysis and cytoplasmic aggregation were also observed. With the increase of time, cinnamaldehyde had no significant difference in the morphological destruction of *E. coli*, which indicated that the treatment of *E. coli* at 2MBC for 3 h had the same effect on the cell membrane and cell contents of *E. coli* as 6, 9, 12, and 24 h.

### Effect of cinnamaldehyde fumigation on the electrical conductivity of the *E. coli* cell suspension

#### Effect of cinnamaldehyde concentration

A permeable barrier of the cell membrane is essential for many cellular functions. Once the cell membrane is damaged, the permeability of the cell is affected, resulting in increased permeability and ion leakage, which increases the EC.

**Figure 3:**
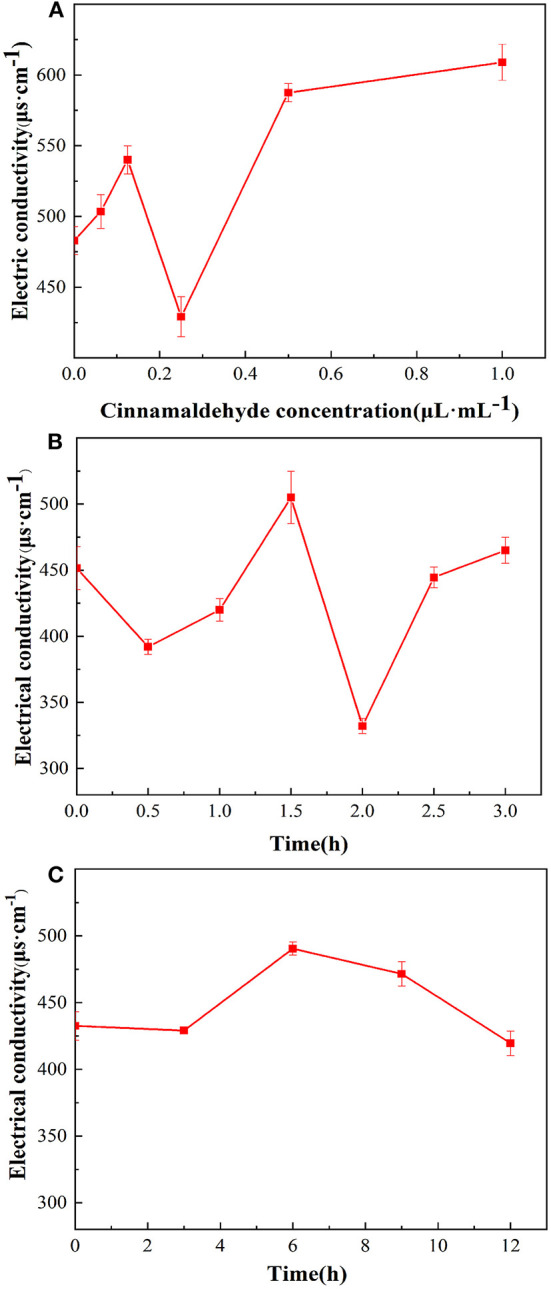
Effect of cinnamaldehyde vapor-phase fumigation of *E. coli* on its electrical conductivity [**(A)** cinnamaldehyde concentration; **(B)** fumigation time (0–3 h); **(C)** fumigation time (0–12 h)].

The changes in the EC of the *E. coli* suspensions fumigated with different concentrations of cinnamaldehyde are presented in Figure 3A. In the range of 0–0.125μL/mL of the cinnamaldehyde concentrations, the EC value increased from 483 to 540 μs/cm, indicating that the fluidity and permeability of the bacterial cell membrane increased after fumigation with cinnamaldehyde. The carrier protein was not denatured at that time, thereby increasing the rate of ions from the intracellular to the bacterial liquid environment, resulting in an increase in the EC value.

In the range of 0.125–0.25 μL/mL of cinnamaldehyde concentration, the EC value decreased to a minimum of 429 μs/cm. This may be because the increased concentration of cinnamaldehyde caused denaturation of the carrier proteins, which restricted diffusion. Therefore, the ions could not cross the cell membrane.

However, with a continued increase in the concentration of cinnamaldehyde, the membrane fluidity decreased, stiffness increased (24), and the membrane was damaged to a certain extent. therefore, intracellular ions could leak directly into extracellular spaces, resulting in an increase in the EC value from 429 to 587 μs/cm. After treatment with cinnamaldehyde at 0.5 μL/mL and above, the EC value of the *E. coli* suspension showed no significant changes. This finding indicated that high concentrations of essential oil severely disrupted the cell membrane structure and severely damaged the cell membrane, resulting in large scale leakage of cellular contents, and eventually the EC reached a stable value.

#### Effect of fumigation treatment time

As shown in Figure 3B that within 3 h of cinnamaldehyde fumigation on *E. coli*, the change in the EC value of the bacterial solution was undulating. It was inferred that cinnamaldehyde was initially adsorbed on the outer surface of the *E. coli* cell membrane, thereby interrupting ionic equilibrium and causing a decrease in the EC value. Then, cinnamaldehyde and the phospholipid layer may have been dissolved, and thus the permeability increased, resulting in the leakage of intracellular ions and an increase in the EC value. After 2 h of treatment, the EC values significantly decreased. This may be because the cinnamaldehyde molecules in the phospholipid layer were saturated and continued to infiltrate the membrane to bind with the membrane lipoproteins. This blocked ion channels reduced the leakage of intracellular contents, and resulted in the corresponding EC values. As time increased, some essential oil molecules penetrated the cell membrane, leading to increased membrane fluidity and a slight increase in the EC value. The change of electrical conductivity after 2.5 h may be due to the fact that cinnamaldehyde had not completely inhibited *E. coli* at the MIC. *E. coli* uses the ions in the system to grow continuously, resulting in no clear change in the EC value of the system.

To verify the trend of conductivity changes at MIC for longer periods, cinnamaldehyde fumigation was performed for 0, 3, 6, 9 and 12 h. As shown in Figure 3C, the changes in conductivity were consistent with those presented in Figure 3B in the initial state and at 3 h. The conductivity changes after 3 h were not significant, indicating that the cell membrane was not completely damaged at the MIC. Although the diffusion of essential oil into the phospholipid bilayer increases the permeability of the cell membrane, the essential oil further penetrates the membrane to bind with lipoproteins, which prevents the transport of ions across the membrane (24, 25). Based on the available literature (24), high concentrations of essential oils lead to a decrease in the proportion of unsaturated fatty acids and an increase in membrane stiffness. These effects caused the EC of the reaction system to maintain a relatively stable value. In summary, at MIC, essential oil fumigation caused changes in the membrane proteins and fatty acids of *E. coli* cell membranes. Moreover, with increasing treatment time, a series of changes occurred, resulting in different cell membrane permeabilities of *E. coli* at different time periods.

### Effect of cinnamaldehyde fumigation on nucleic acid leakage in *E. coli*

#### Effect of cinnamaldehyde concentration

When the cell membrane of *E. coli* is disrupted, intracellular macromolecules such as nucleic acids and proteins leak out of the membrane. Several studies have shown that severe nucleic acid leakage occurs after treatment with plant-based essential oils (26, 27).

**Figure 4:**
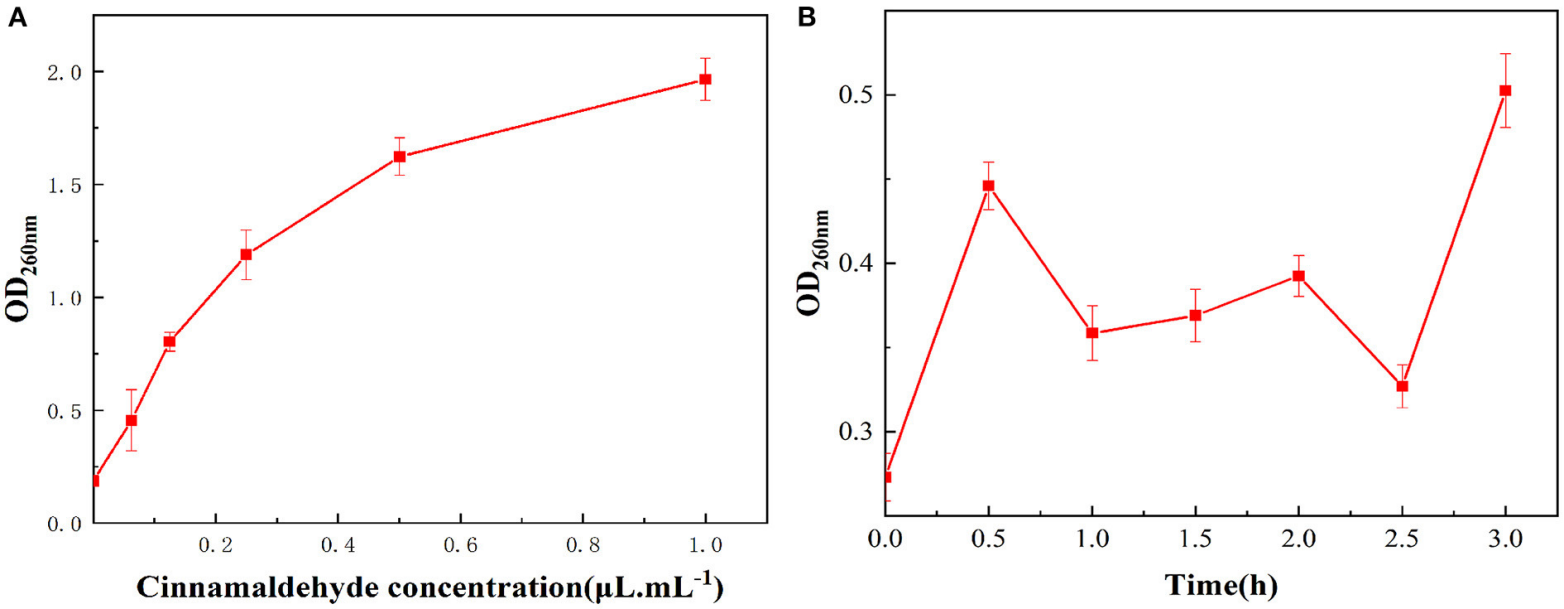
Effect of vapor-phase cinnamaldehyde fumigation of *E. coli* on nucleic acid leakage [**(A)** cinnamaldehyde concentration; **(B)** fumigation time].

As the cinnamaldehyde fumigation concentration increased shown in Figure 4A, the absorbance value of the *E. coli* extracellular fluid at 260 nm also increased, indicating that OD_260nm_ was positively correlated with the concentration of cinnamaldehyde, which was consistent with a previous study (28). It was speculated that after fumigation with cinnamaldehyde, the cell membrane of *E. coli* was damaged, and the degree of damage increased with increasing concentrations of cinnamaldehyde, resulting in the leakage of nucleic acid from the intracellular fluid to the extracellular fluid and an increase in absorbance at 260 nm.

#### Effect of fumigation treatment time

The results of the OD_260nm_ measurements of the bacterial suspension of *E. coli* fumigated with the MIC of cinnamaldehyde at different times are shown in Figure 4B. With the extension of cinnamaldehyde fumigation time, the absorbance values of the *E. coli* extracellular fluid at 260 nm within 3 h fluctuated within a small range, which was higher than that of the blank. This finding indicated that at the MIC of cinnamaldehyde, fumigation for 3 h could cause damage to the cell membrane, leading to the leakage of its nucleic acids. However, within the time frame of this experiment, the MIC was insufficient to cause significant effects on the *E. coli* cell membrane. Comparing Figures 4A,B, the OD_260nm_ values after 24 h fumigation were significantly higher than 3 h fumigation at the MIC. Accordingly, with the extension of fumigation time, the overall trend of OD_260nm_ values increased within 24 h. Moreover, fumigation time was positively correlated with cell membrane damage.

### Effect of cinnamaldehyde fumigation damage on the structure of *E. coli* membrane proteins

#### Effect on the tertiary structure of *E. coli* membrane proteins

**Figure 5:**
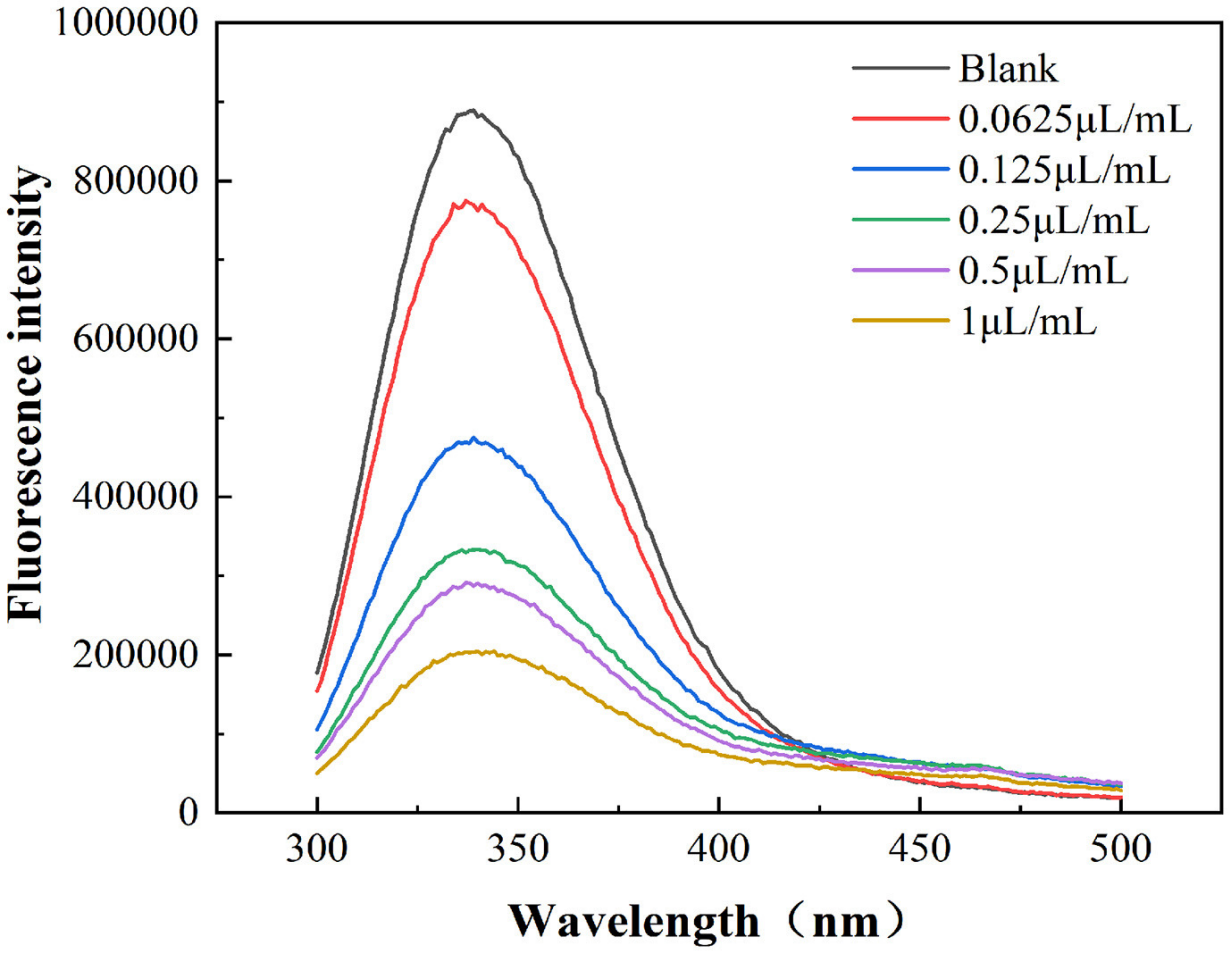
Fluorescence emission spectra of *E. coli* treated by different concentrations of cinnamaldehyde.

As shown in Figure 5, the wavelength of the maximum absorbance peaks of the *E. coli* membrane proteins remained unchanged after fumigation with different cinnamaldehyde concentrations. However, the fluorescence intensity exhibited a regular change. With a continuous increase in the concentration of cinnamaldehyde, the fluorescence intensity of the maximum absorbance peak became weaker, indicating that the fluorescence of the *E. coli* membrane proteins was quenched by cinnamaldehyde fumigation. This may be due to the coiling and folding of *E. coli* membrane proteins, which reduces the exposure of the chromogenic groups and thereby reduces the fluorescence intensity (22). The results suggested that the lethal effects of cinnamaldehyde fumigation on *E. coli* may be affecting and denaturing *E. coli* membrane proteins, and then affecting the original functions of protein, ultimately leading cell to death.

#### Effect on the secondary structure of *E. coli* membrane proteins

**Figure 6:**
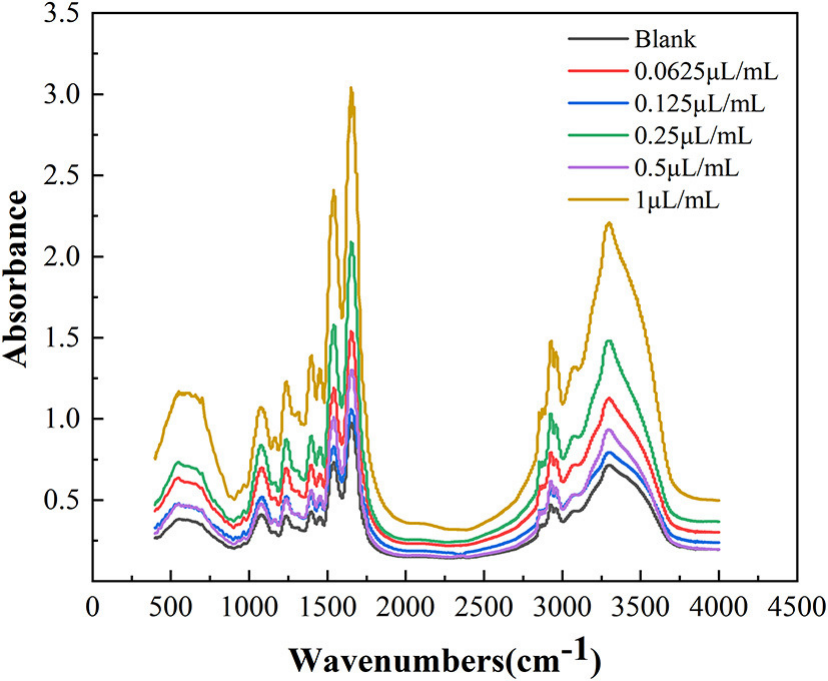
FTIR spectra of *E. coli* with different concentration cinnamaldehyde.

Infrared spectroscopy can effectively elucidate conformational information of the secondary structure of proteins. As shown in Figure 6, the *E. coli* membrane proteins had strong absorbance peaks in the amide I band (1,600–1,700 cm^−1^), with differences in the characteristic absorbance peaks of different cinnamaldehyde fumigated *E. coli* proteins.

**Figure 7:**
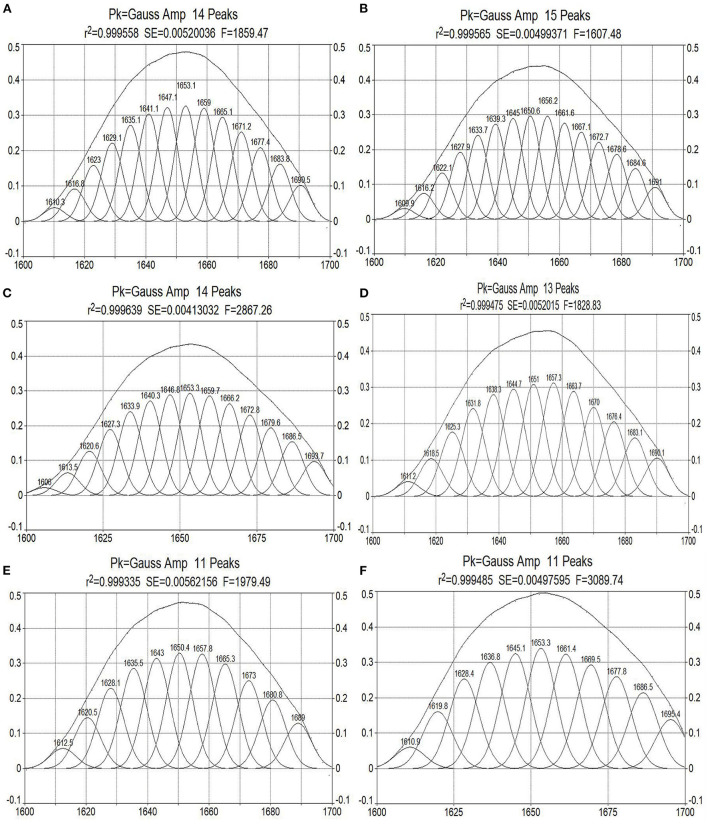
Curve-fitted results of amide I bands of *E. coli* treated by cinnamaldehyde with different concentration [**(A–F)** are Blank, 0.0625, 0.125, 0.25, 0.5, and 1.0 μL/mL of cinnamaldehyde fumigation].

The amide I band of the bacterial infrared spectrum is dominated by the absorption of the stretching vibrations of the C=O bond of its amino acid residues, which is mainly reflected by the helix, sheets, turns, and random coil structures of the proteins. To determine the effect of cinnamaldehyde on the secondary structure of the membrane protein, the amide I band was processed using a second-order derivative fitting to obtain the subpeaks, as shown in Figure 7. The subpeaks and their corresponding secondary structures were as follows: 1,700–1,680 cm^−1^ for antiparallel β-sheets, 1,680–1,660 cm^−1^ for β-turns, 1,660–1,650 cm^−1^ for α-helix, 1,650–1,640 cm^−1^ for random coils, and 1,640–1,610 cm^−1^ for β-sheets. The density of the protein molecules is embodied in the *α-*helix structure, whereas other structures, such as *β-*sheets, turns, and random coils reflect the looseness of the protein molecule (20). The calculated results are listed in Table 2.

**Table 2:** The contents of secondary structure of *E. coli* treated by different concentration of cinnamaldehyde.

**Concentration/** **(μL·mL^−1^)**	**Contents of secondary structure (%)**			
	**α-helix**	**β-sheets**	**β-turns**	**Random coils**
0	21.01	25.47	33.17	20.35
0.0625	9.96	30.83	39.47	19.74
0.125	21.44	33.06	34.80	10.70
0.25	22.42	30.52	36.38	10.68
0.5	25.61	28.01	34.10	12.28
1.0	12.73	29.04	46.06	12.17

As shown in Table 2, with increasing concentrations of cinnamaldehyde fumigation, α-helix showed an undulating change pattern. β-turns and β-sheets showed similar trends, with the contents treated with cinnamaldehyde being higher than that of the blank. The random coil content decreased with the fumigation of cinnamaldehyde.

The results suggested that the polypeptide chains of membrane proteins tended to change from random coils to linear and helical structures after cinnamaldehyde fumigation, so that the exposed chromophoric groups were covered, which is consistent with the previous results of weakened fluorescence intensity. This reflected the compactness of the *E. coli* protein molecules after fumigation with vapor-phase cinnamaldehyde.

## Conclusion

Since there has been no standard methods exist to evaluate the antimicrobial activity in vapor phase (13), different methods have been used by different authors. The values of MIC and MBC in the paper did not necessarily represent the actual cinnamaldehyde vapor concentration, because of leakage outside, absorption into the medium. For this reason, the concentrations in vapor phase should be considered as indicative values only (13, 29). If required, the exact concentrations can be determined e.g., using headspace analysis. However, the values of MIC and MBC were reproducible when the assay conditions were identical, and allowed comparison of bioactivity of different cinnamaldehyde concentration in the study.

37°C is the optimum temperature for *E. coli* to grow, which was chosen to better reflect how well the bacteria had been inhibited or killed. But many researchers demonstrated that the antimicrobial activity of essential oils and volatile molecules depended on their vapor pressure in the head space and its evolution over time (30, 31), the factors such as temperature is able to regulate the vapor pressure of the essential oils and raise antimicrobial activity. When conducting application experiments in the future work, comparative experiments at 37°C and food storage temperatures will be considered.

In summary, we successfully developed a promising strategy for investigated the antimicrobial activity and mechanism of action of vapor-phase cinnamaldehyde against *E. coli*. In this study, the MIC of cinnamaldehyde vapor-phase fumigation on *E. coli* was 0.25 μL/mL and the MBC was 0.5 μL/mL. Cinnamaldehyde disrupts the morphology of *E. coli* cells, causing cell surface ruffling, cytoplasmic aggregation, and plasmolysis. At cinnamaldehyde concentrations lower than the MBC, the changes in the conductivity of the bacterial suspension might be related to the fatty acid composition and the degree of cell membrane protein binding. Furthermore, the permeability of the *E. coli* cell membrane differed at different time periods within 12 h of fumigation at the MIC. At concentrations above the MBC, cinnamaldehyde severely disrupted the cell membrane and the conductivity of the bacterial suspension remained stable. The OD_260nm_ value was positively correlated with the concentration of cinnamaldehyde, indicating that cinnamaldehyde caused damage to the cell membrane, thereby resulting in leakage of biological macromolecules. The fluorescence intensity of the maximum absorption peak of the *E. coli* suspension was found to be negatively correlated with the concentration of cinnamaldehyde, indicating that cinnamaldehyde causes changes in the tertiary structure of membrane proteins. With increasing concentrations of cinnamaldehyde, the bacterial membrane proteins tended to curl and fold. This study provides a theoretical basis for the study of the antibacterial effects of vapor-phase cinnamaldehyde and elucidates the mechanism of the antibacterial activity of cinnamaldehyde vapor-phase fumigation against *E. coli*. This study provides an important approach for studying the inhibitory activity and mechanisms of plant essential oils on microorganisms.

More recently, essential oils are often added to food packaging due to their vapor-phase antimicrobial activity. One way is to directly use the essential oil fragrance to form an antibacterial atmosphere, and the other way is to add essential oils to the film to make an active packaging film, which can release antibacterial and keep food fresh. Sun et al. (32) examined the antifungal efficiency of cinnamaldehyde against *Aspergillus niger in vitro*, and evaluated its application potential in bread preservation *in situ*. The result showed that, in the vapor phase, cinnamaldehyde can efficiently prolong the shelf life of bread. Nutchanat et al. (33) achieved fabrication of an antibacterial gelatin-bacterial cellulose nanocomposite film using cinnamaldehyde as an antibacterial additive and a crosslinker, the films showed strong antibacterial activity against *E. coli* and *S. aureus*, which is proposed to be a good candidate for an antibacterial food packaging. Cui et al. (34) loaded cinnamaldehyde on etched halloysite nanotubes and adding it to sodium alginate matrix to prepare antimicrobial food packaging film. The result showed that the film containing cinnamaldehyde had a good antibacterial activity against *E. coli* and *S. aureus*, and the firm had a controlled release effect on cinnamaldehyde. Thus, essential oils could potentially be applied to the food packaging industry as active packaging materials in the non-direct contact with food.

## Data availability statement

The raw data supporting the conclusions of this article will be made available by the authors, without undue reservation.

## Author contributions

XD designed and wrote the manuscript. DQ, HL, TZ, and YHu provided the data. XD and TZ analyzed the data. YHa, DH, KW, XC, and CC revised the manuscript. All authors listed have approved it for publication, contributed to the article, and approved the submitted version.

## Funding

This work was supported by grant from the Forestry Scientific Technology Innovation Project of Guangdong Province (2020KJCX010), Science & Technology Planning Project of Guangzhou City (202103000078 and 202206010181), Science & Technology Planning Project of Guangdong Province (19ZK0364), and College Student Innovation and Entrepreneurship Training Program of Guangdong University of Technology (202011845016 and S202111845115).

## Conflict of interest

The authors declare that the research was conducted in the absence of any commercial or financial relationships that could be construed as a potential conflict of interest.

## Publisher's note

All claims expressed in this article are solely those of the authors and do not necessarily represent those of their affiliated organizations, or those of the publisher, the editors and the reviewers. Any product that may be evaluated in this article, or claim that may be made by its manufacturer, is not guaranteed or endorsed by the publisher.

## References

[1] Ning YW Su D Yu YN Yang YL Wang ZX Ying YM. Antibacterial mechanism of antimicrobial peptide brevilaterin combined with citric acid against *Escherichia coli*. Food Sci. (2020) 41:31–7. 10.7506/spkx1002-6630-20191008-026

[2] Mughini-Gras L Van Pelt W Van der Voort M Heck M Friesema I Franz E. Attribution of human infections with Shiga toxin-producing *Escherichia coli* (STEC) to livestock sources and identification of source-specific risk factors, The Netherlands (2010-2014). Zoonoses Public Health. (2017) 65:8–22. 10.1111/zph.12403 28921940

[3] Duan HY Chai TJ Cai YM Zhong ZB Yao ML Zhang XX. Transmission identification of *Escherichia coli* aerosol in chicken houses to their environments using ERIC-PCR. Sci China Ser C Life Sci. (2008) 51:164–73. 10.1007/s11427-008-0021-0 18239895 PMC7089447

[4] Ci Y Wang S Wang J Zhang XL. Research progress on botanical antibacterial disinfectant. Chin J Front Health Quar. (2020) 43:297–300. 10.16408/j.1004-9770.2020.04.022

[5] Yin LZ Chen JH Wang KY Geng Y Lai WM Huang XL. Study the antibacterial mechanism of cinnamaldehyde against drug-resistant *Aeromonas hydrophila* in vitro. Microb Pathog. (2020) 145:104208. 10.1016/j.micpath.2020.104208 32325237

[6] Mooyottu S Kollanoor-Johny A Flock G Bouillaut L Upadhyay A Sonenshein A. Carvacrol and trans-cinnamaldehyde reduce clostridium difficile toxin production and cytotoxicity in vitro. Int J Mol Sci. (2014) 15:4415–30. 10.3390/ijms15034415 24625665 PMC3975404

[7] Wang LH Wang MS Zeng XA Gong DM Huang YB. An in vitro investigation of the inhibitory mechanism of β-galactosidase by cinnamaldehyde alone and in combination with carvacrol and thymol. Biochim Biophys Acta Gen Subj. (2017) 1861:3189–98. 10.1016/j.bbagen.2016.08.002 27531708

[8] He TF Zhang ZH Zeng XA Wang LH Brennan CS. Determination of membrane disruption and genomic DNA binding of cinnamaldehyde to *Escherichia coli* by use of microbiological and spectroscopic techniques. J Photochem Photobiol B. (2018) 178:623–30. 10.1016/j.jphotobiol.2017.11.015 29306845

[9] Ye HQ Shen SX Xu JY Lin SY Yuan Y Jones GS. Synergistic interactions of cinnamaldehyde in combination with carvacrol against food-borne bacteria. Food Control. (2013) 34:619–23. 10.1016/j.foodcont.2013.05.032

[10] Kot B Wierzchowska K Gruzewska A Lohinau D. The effects of selected phytochemicals on biofilm formed by five methicillin-resistant *Staphylococcus aureus*. Nat Prod Res. (2017) 32:1299–302. 10.1080/14786419.2017.1340282 28627304

[11] Inouye S Takizawa T Yamaguchi H. Antibacterial activity of essential oils and their major constituents against respiratory tract pathogens by gaseous contact. J Antimicrob Chemother. (2001) 47:565–73. 10.1093/jac/47.5.565 11328766

[12] Yun J Fan XT Li XH. Inactivation of *Salmonella entericaserovar Typhimurium* and quality maintenance of cherry tomatoes treated with gaseous essential oils. J Food Sci. (2013) 78:458–64. 10.1111/1750-3841.12052 23398191

[13] Houdkova M Rondevaldova J Doskocil I Kokoska L. Evaluation of antibacterial potential and toxicity of plant volatile compounds using new broth microdilution volatilization method and modified MTT assay. Fitoterapia. (2017) 118:56–62. 10.1016/j.fitote.2017.02.008 28223069

[14] OuYang QL Okwong RO Chen YP Tao NG. Synergistic activity of cinnamaldehyde and citronellal against green mold in citrus fruit. Postharvest Biol Technol. (2020) 162:111095. 10.1016/j.postharvbio.2019.111095

[15] Hernández-Herrero LA Giner MJ Valero M. Effective chemical control of psychrotrophic *Bacillus cereus* EPSO-35AS and INRA TZ415 spore outgrowth in carrot broth. Food Microbiol. (2008) 25:714–21. 10.1016/j.fm.2008.02.004 18541171

[16] Wang F Yang JD Wang CM Shi ZQ. Inhibitory effect of cinnamaldehyde against *Escherichia coli* and *Pseudo-monas aeruginosa*. Jiangsu J Agr Sci. (2011) 27:888–92. 10.1007/s11676-011-0141-4

[17] Rogiers G Kebede BT Van Loey A Michiels CW. Membrane fatty acid composition as a determinant of *Listeria monocytogenes* sensitivity to trans-cinnamaldehyde. Res Microbiol. (2017) 168:536–46. 10.1016/j.resmic.2017.03.001 28342836

[18] Li TT Wang DF Liu N Ma Y Ding T Mei YC. Inhibition of quorum sensing-controlled virulence factors and biofilm formation in Pseudomonas fluorescens by cinnamaldehyde. Int J Food Microbiol. (2018) 269:98–106. 10.1016/j.ijfoodmicro.2018.01.023 29421365

[19] Sharma G Raturi K Dang S Gupta S Gabrani R. Inhibitory effect of cinnamaldehyde alone and in combination with thymol, eugenol and thymoquinone against *Staphylococcus epidermidis*. J Herb Med. (2017) 9:68–73. 10.1016/j.hermed.2016.11.001

[20] Duan XJ Han YL Liu ZX Zhang T Xu YT Huang YQ. Antibacterial mechanism of cinnamon essential oil vapor fumigation against *Staphylococcus aureus*. Mod Food Sci Technol. (2021) 37:50–8. 10.13982/j.mfst.1673-9078.2021.9.1210

[21] Wu KG Zhao XX Chai XH Yu HP Liu XL Fan YT. Antibacterial activity and mechanism of action of vapor-phase linalool. Food Sci. (2020) 41:61–7. 10.7506/spkx1002-6630-20181130-365

[22] Wu KG Lin YH Chai XH Duan XJ Zhao XX Chun C. Mechanisms of vapor-phase antibacterial action of essential oil from cinnamomum camphora var. linaloofera Fujita against *Escherichia coli*. Food Sci Nutr. (2019) 7:2546–55. 10.1002/fsn3.1104 31428342 PMC6694428

[23] Paul S Dubey RC Maheswari DK Kang SC. Trachyspermum ammi (L) fruit essential oil influencing on membrane permeability and surface characteristics in inhibiting food-borne pathogens. Food Control. (2011) 22:725–31. 10.1016/j.foodcont.2010.11.003

[24] Sarengaowa H Feng K Xiu ZL Jiang AL Lao Y. Antimicrobial mechanisms of essential oils and their components on pathogenic bacteria: a review. Food Sci. (2020) 41:285–94. 10.7506/spkx1002-6630-20190603-018

[25] Li YR Zhou LY Li SR Cao ZZ Zhang L Wei M. Antibacterial activity and mechanism of action of plant essential oils and their main components from fruits and vegetables: a review. Food Sci. (2014) 35:325–9. 10.7506/spkx1002-6630-201411063

[26] Kang JM Jin WY Wang JF Sun YY Wu XX Liu L. Antibacterial and anti-biofilm activities of peppermint essential oil against S*taphylococcus aureus*. LWT-Food Sci Technol. (2019) 101:639–45. 10.1016/j.lwt.2018.11.093

[27] Ziaee E Razmjooei M Shad E Eskandari MH. Antibacterial mechanisms of Zataria multiflora Boiss. essential oil against *Lactobacillus curvatus*. LWT-Food Sci Technol. (2018) 87:406–12. 10.1016/j.lwt.2017.08.089

[28] Hu W Li CZ Dai JM Cui HY Lin L. Antibacterial activity and mechanism of Litsea cubeba essential oil against methicillin-resistant *Staphylococcus aureus* (MRSA). Ind Crops Prod. (2019) 130:34–41. 10.1016/j.indcrop.2018.12.078

[29] Houdkova M Kokoska L. Volatile antimicrobial agents and in vitro methods for evaluating their activity in the vapour phase: a review. Planta Med. (2020) 86:822–57. 10.1055/a-1158-4529 32450573

[30] Tyagi AK Malik A Gottardi D Guerzoni ME. Essential oil vapour and negative air ions: a novel tool for food preservation. Trends Food Sci Tech. (2012) 26:99–113. 10.1016/j.tifs.2012.02.004

[31] Gardini F Lanciotti R Guerzoni ME. Effect of trans-2-hexenal on the growth of *Aspergillus flavus* in relation to its concentration, temperature and water activity. Lett Appl Microbio. (2001) 33:50–5. 10.1046/j.1472-765X.2001.00956.x 11442815

[32] Sun Q Li JM Sun Y Chen Q Zhang L Le T. The antifungal effects of cinnamaldehyde against *Aspergillus niger* and its application in bread preservation. Food Chem. (2020) 317:126405. 10.1016/j.foodchem.2020.126405 32078995

[33] Nutchanat A Siriporn T Malinee S Suchata K. Antibacterial activity in gelatin-bacterial cellulose composite film by thermally crosslinking with cinnamaldehyde towards food packaging application. Food Pack Shelf. (2022) 31:100766. 10.1016/j.fpsl.2021.100766

[34] Cui R Zhu BF Yan JT Qin YY Yuan MW Cheng GG. Development of a sodium alginate-based active package with controlled release of cinnamaldehyde loaded on halloysite nanotubes. Foods. (2021) 10:1150. 10.3390/foods10061150 34063767 PMC8223774

